# Dependability of Procalcitonin as an Early Predictor of Infection in Open Fractures: An Observational Study

**DOI:** 10.7759/cureus.75892

**Published:** 2024-12-17

**Authors:** Alden D Souza, Dileep K. S.

**Affiliations:** 1 Department of Orthopaedics, K S Hegde Medical Academy, Mangaluru, IND

**Keywords:** infection prediction, open fractures, orthopedic trauma, postoperative infection, procalcitonin (pct)

## Abstract

Introduction

Open extremity fractures are complex injuries involving soft tissue disruption and bone discontinuity, often associated with significant morbidity and mortality due to complications such as infection. Infection remains a primary concern, exacerbating patient outcomes and increasing healthcare costs. Procalcitonin (PCT) is a biomarker with potential utility for early detection of infection in these cases.

Materials and methods

This prospective observational study was conducted at Justice K S Hegde Charitable Hospital between October 2022 and April 2024. Forty patients with open fractures who met inclusion criteria were enrolled. PCT levels were measured preoperatively and on postoperative days 1 and 5. Patients were monitored for clinical signs of infection for one month. Data analysis included descriptive and inferential statistics.

Results

The mean age of the patients was 42.26 ± 16.62 years, with a male predominance of 35 (87.5%) of the total sample size. Preoperative PCT levels were significantly higher in patients who developed infections (mean: 1.02 ng/mL) compared to non-infected patients (mean: 0.13 ng/mL). Postoperative PCT levels continued to rise in all patients but were significantly elevated in the infected group (p < 0.01).

Conclusion

PCT is a reliable biomarker for the early detection of postoperative infections in open fractures. Elevated preoperative PCT levels (>0.5 ng/mL) predict infection, while non-infected patients showed lower trauma-related increases. Further studies with larger samples are recommended to validate these findings.

## Introduction

Open extremity fractures are severe injuries characterised by disruption of the surrounding soft tissues and loss of bone continuity. The severity of these injuries depends on factors such as the velocity of trauma, degree of contamination, and extent of tissue damage, which together contribute to a significant risk of morbidity and mortality [1]. Globally, open fractures represent a major epidemiological challenge. The complexity of managing open fractures arises from the need to address additional soft tissue damage caused by the open wound, which exposes the fracture hematoma to contamination [2]. Infection is one of the most common complications associated with these injuries [3]. According to findings from the Lower Extremity Assessment Project (LEAP) study, 3.9% of patients had unhealed wounds and 10.9% experienced non-union of fractures even two years post-injury [4]. Patients with open fractures frequently experience a significantly diminished quality of life in the initial stages, which can severely impact their mental well-being [5].

While it is widely acknowledged that specialists should treat severe open fractures, emergency care may often involve general surgeons. Therefore, all surgeons must have a basic understanding of managing these injuries [6]. Infection prevention is a cornerstone of open fracture treatment. The risk of infection is primarily influenced by factors such as bacterial load, pathogen virulence, and host susceptibility. Conditions such as shock, localised haemorrhage, dead space, fracture instability, lack of viable tissue, and systemic comorbidities, including diabetes, immunosuppression, and ischemia, exacerbate the likelihood of infection [7]. In developed nations, infections are increasingly caused by hospital-acquired pathogens, whereas in resource-limited settings, delays in access to modern medical care remain a critical issue [7,8]. Effective management of these injuries includes early skeletal stabilisation and soft tissue coverage, which are essential for achieving favourable outcomes [9,10]. The Gustilo-Anderson Classification System (GACS) is the most widely used framework for classifying open fractures and guiding therapeutic decision-making [10].

Biomarkers such as C-reactive protein (CRP), white blood cell count (WBC), and erythrocyte sedimentation rate (ESR) are commonly used in clinical settings to detect early infections in open fractures. However, these markers often lack specificity for sepsis. CRP, for instance, is not a reliable indicator of systemic inflammation in open fractures [11]. Procalcitonin (PCT), a prohormone of calcitonin produced by neuroendocrine medullary C-cells of the thyroid gland, is a more specific biomarker for bacterial infections. In healthy individuals, circulating PCT levels are low (<0.05 ng/mL). During bacterial infections, PCT levels increase due to cytokine stimulation such as IL-1, tumour necrosis factor-α, IL-6, and bacterial endotoxins [11]. Unlike CRP, which begins to rise 12-24 hours post-injury and peaks at 48 hours, PCT levels increase within two to four hours and peak between six and 24 hours [12].

Additionally, PCT production is unaffected by immunosuppressive conditions such as neutropenia, and higher PCT levels correlate with the severity of bacterial infections [13]. Elevated PCT concentrations are associated with a greater mortality risk, underscoring its predictive value [14]. Compared to other biomarkers, PCT offers advantages due to its specificity for bacterial infections, rapid response to infection, strong correlation with disease severity, and resilience to immunosuppressive conditions [15].

Given these attributes, the present study aims to evaluate the reliability of PCT as an early predictor of infection in open fractures.

## Materials and methods

Study setting

This prospective observational study was conducted in the Department of Orthopaedics at Justice K S Hegde Charitable Hospital, affiliated with K S Hegde Medical Academy, Nitte University, Deralakatte, Mangaluru - 575018. The hospital-based study spanned from October 1, 2022, to April 30, 2024, and included all patients presenting to the hospital during this period.

Study participants

A sample size of 40 participants was determined using nMaster Software Version 2 (Department of Biostatistics at Christian Medical College, Vellore, India), based on a preoperative PCT standard deviation of 19.2 pg/mL as per the study of Saleh et al. [16], with a 5% level of significance and a margin of error of 6.

Inclusion criteria

It includes patients presenting with open fractures within 24 hours of trauma and undergoing internal fixation within three to 40 hours post-trauma.

Exclusion criteria

The exclusion criteria were patients with pre-existing clinically detectable infections such as urinary tract infections or pneumonia.

Study procedure

Patients with open fractures during the study period were evaluated using standard radiographs, and PCT levels were measured preoperatively and on postoperative days 1 and 5. Following surgery, patients were monitored for signs of infection, including wound discharge, fever, local induration, gaping of the wound, and bacterial growth on wound culture over one month. PCT levels were quantified using an immunometric immunoassay (VITROS Biological Research and Applied Molecular Medicine System (B.R.A.H.M.S) PCT), an established laboratory method. Data accompanied by informed consent were recorded for patients who met the inclusion criteria.

Ethical consideration

This study was approved by the Institutional Ethics Committee, K S Hegde Medical Academy, Mangaluru, Karnataka, on August 8, 2022, under expedited review with Institutional Ethics Committee letter number EC/EC/170/2022.

Statistical analysis

Statistical analysis was performed using SPSS version 29 (IBM SPSS Statistics for Windows, IBM Corp., Armonk, NY). Descriptive statistics were computed for explanatory and outcome variables, using means and standard deviations for quantitative data and frequencies and proportions for qualitative data. Inferential statistics included repeated measures ANOVA to compare PCT levels at different time intervals, with post hoc least significant difference (LSD) tests for comparisons between intervals. The significance level was set at 5%.

## Results

The mean age of patients was 42.26 ± 16.62 years. Five (12.5%) were females, and 35 (87.5%) were males. The cause of injury was explosive in one (2.5%), road traffic accident (RTA) in 21 (52.5%), fall from height in seven (17.5%), machine cut injury in five (12.5%), fall of heavy object in four (10%), assault in one (2.5%) and sickle cut injury in one (2.5%). Femur and tibia were involved in two (5%), tibia and fibula in eight (20%), tibia in 14 (35%), femur in four (10%), radius and ulna in one (2.5%), phalanx in seven (17.5%), metatarsal in two (5%), ulna in one (2.5%), calcaneum in 1 (2.5%). Gustilo-Anderson (GA) type 1 fracture was present in three (7.5%), GA type 2 fracture was present in four (10%), GA type 3A fracture was present in seven (17.5%), GA type 3B fracture was present in 18 (45%), and GA type 3C fracture was present in 9 (22.5%) (Table 1).

**Table 1:** Baseline characteristics

Parameter	Count frequency (n)	Percentage (%)
Gender		
Female patients	5	12.5
Male patients	35	87.5
Type of injury		
Explosive injury	1	2.5
Road traffic accident	21	52.5
Fall from height	7	17.5
Machine cut injury	5	12.5
Fall of heavy object	4	10
Assault	1	2.5
Sickle cut injury	1	2.5
Bone involved		
Femur and tibia	2	5
Tibia and fibula	8	20
Tibia	14	35
Femur	4	10
Radius and ulna	1	2.5
Phalanx	7	17.5
Metatarsal	2	5
Ulna	1	2.5
Calcaneum	1	2.5
Type of fracture		
GA type 1 fracture	3	7.5
GA type 2 fracture	4	10
GA type 3A fracture	7	17.5
GA type 3B fracture	18	45
GA type 3C fracture	9	22.5

**Figure 1:**
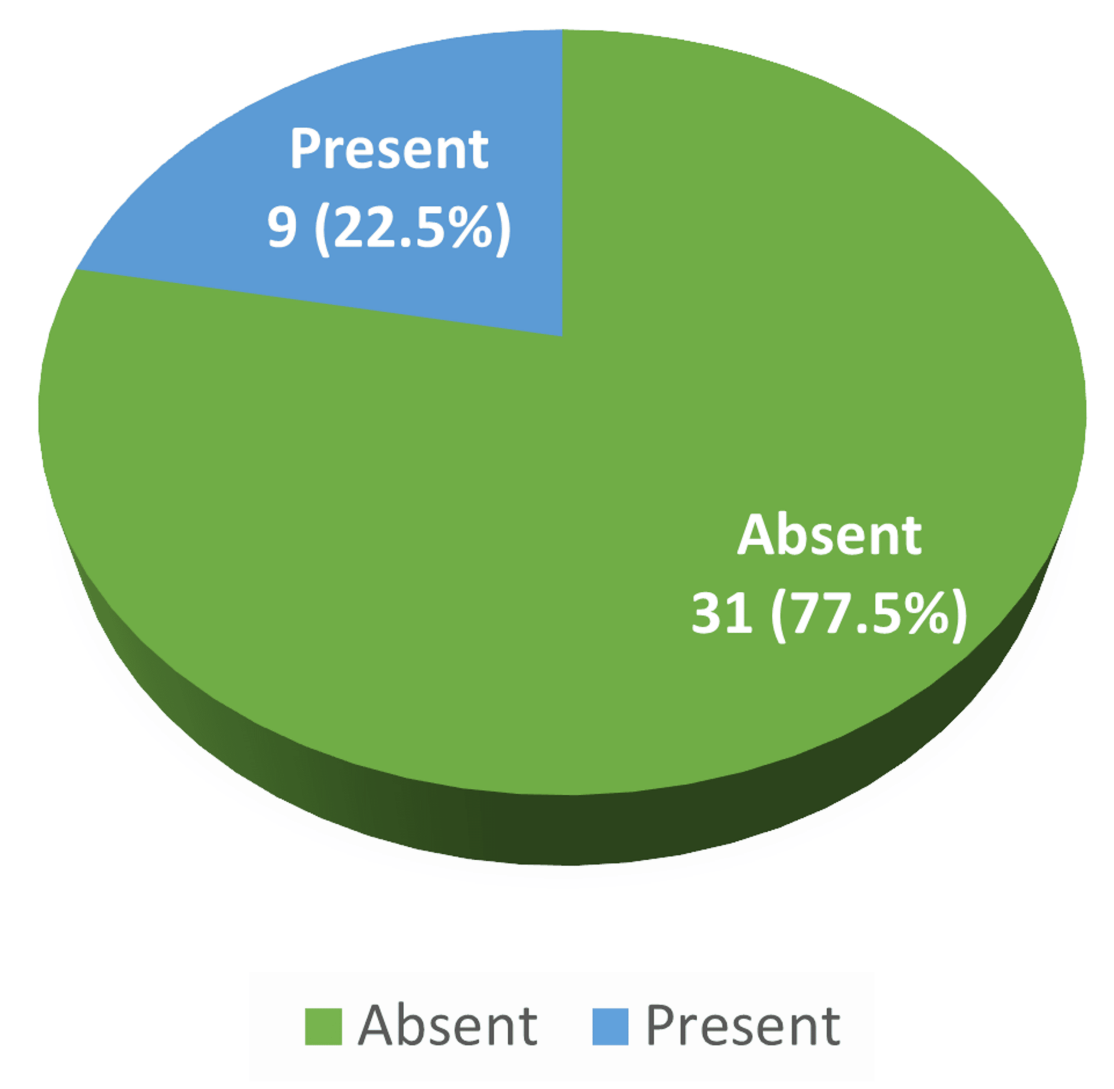
Distribution of subjects based on wound infection

Wound infection was present in nine (22.5%) (Figure 1).

**Figure 2:**
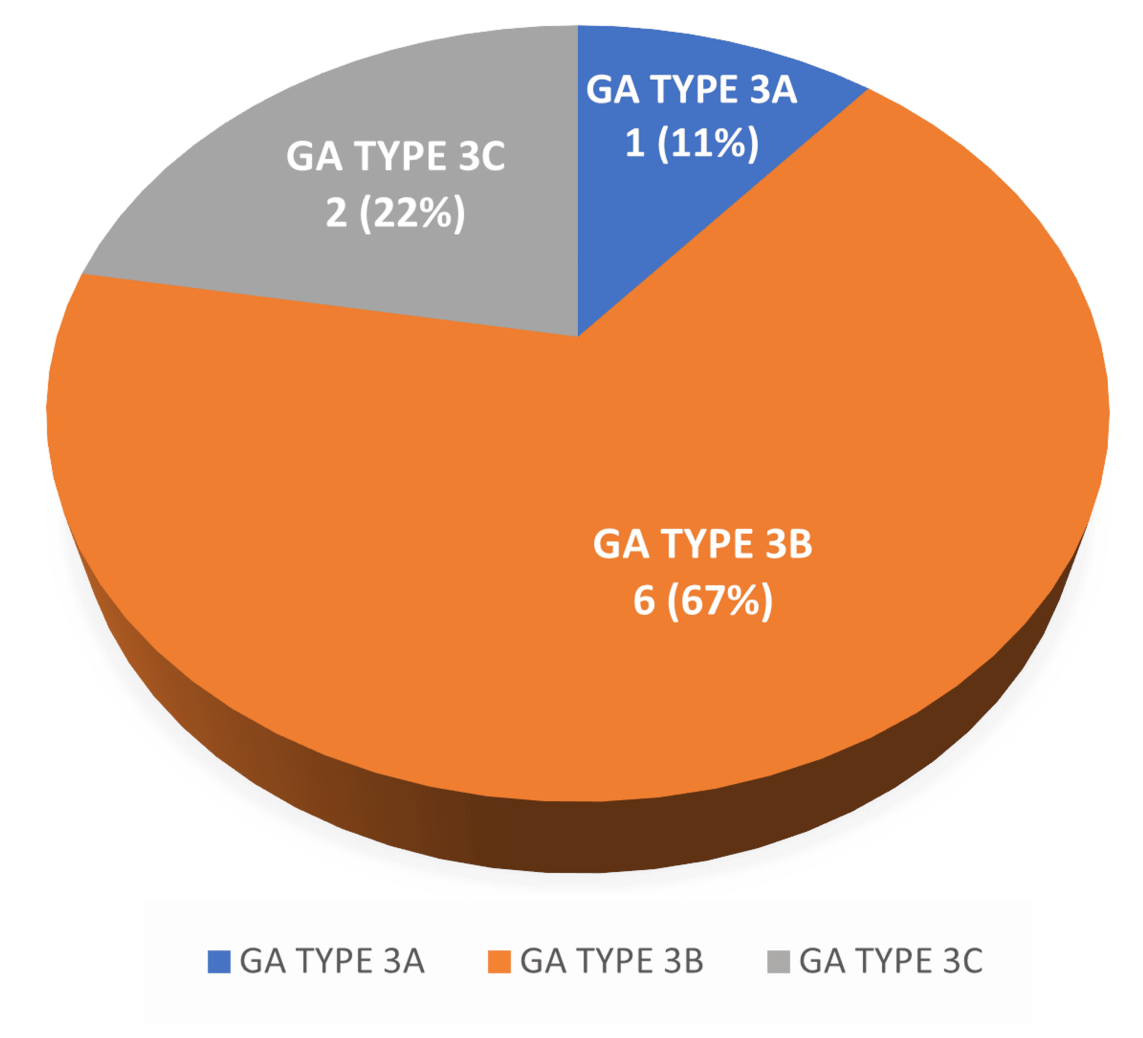
Incidence of infection in the type of open fracture

Infection in GA type 3A fracture was seen in one (11.1%), GA type 3B fracture was seen in six (66.7%), and GA type 3C fracture was seen in two (22.2%) (Figure 2).

*Escherichia coli *and *Enterococcus faecalis* were isolated in two (22.2%), *Staphylococcus aureus* was isolated in one (11.1%), *Enterobacter hormaechei* was isolated in one (11.1%), *Escherichia coli* and *Serratia marcescens* was isolated in one (11.1%), *Pseudomonas aeruginosa* was isolated in 1 (11.1%), and no growth was seen in three (33.3%) (Table 2).

**Table 2:** Culture reports of postoperative infected wounds

Organism growth	Frequency	Percentage (%)
*Escherichia coli* and *Enterococcus faecalis*	2	22.2
Staphylococcus aureus	1	11.1
Enterobacter hormaechei	1	11.1
*Escherichia coli* and *Serratia marcescens*	1	11.1
Pseudomonas aeruginosa	1	11.1
No growth	3	33.3

**Figure 3:**
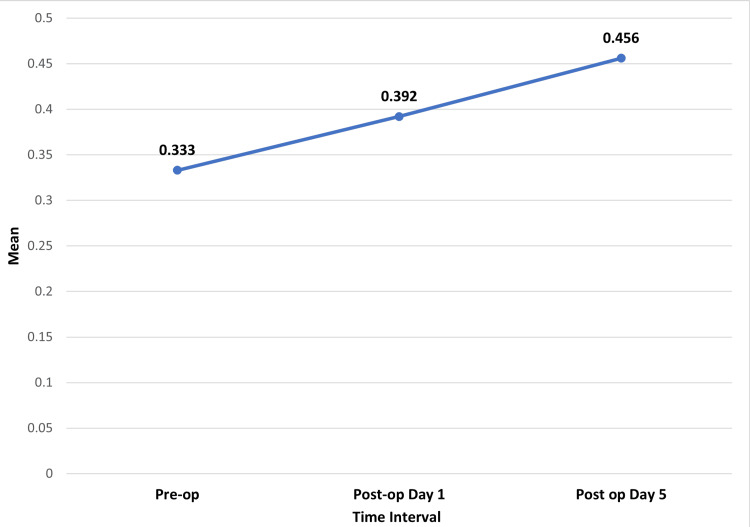
Mean procalcitonin levels at different time intervals

The mean preoperative PCT level was 0.33 ± 0.46. The mean postoperative day 1 PCT level was 0.39 ± 0.46. The mean postoperative day 5 PCT level was 0.45 ± 0.53. The comparison between mean PCT levels at different time intervals was statistically significant in the present study (p = 0.01) (Figure 3).

The preoperative vs. postoperative day 1 PCT level was statistically significant (p = 0.006). The preoperative vs. postoperative day 5 PCT level was statistically significant (p = 0.008). There was no statistically significant difference between PCT levels at postoperative day 1 vs. day 5 (p = 0.132) (Table 3).

**Table 3:** Multiple comparisons of the procalcitonin levels between different time intervals using post hoc least significant difference (LSD) *Significant (p < 0.05)

	Mean difference	p-value
Preoperative vs. postoperative day 1	-0.058	0.006*
Preoperative vs. postoperative day 5	-0.122	0.008*
Postoperative day 1 vs. day 5	-0.064	0.132

**Figure 4:**
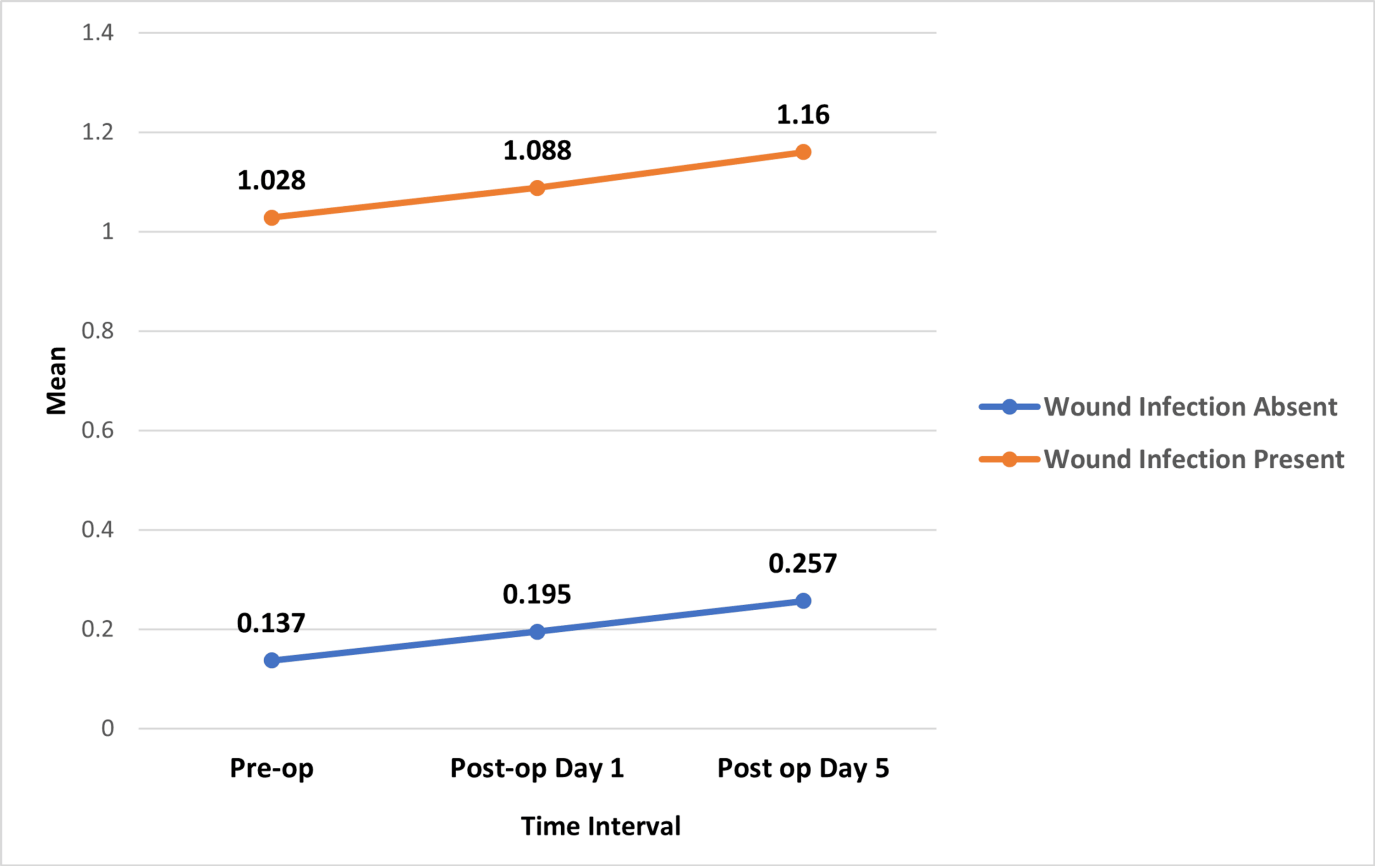
Mean procalcitonin levels at different time intervals (non-infected vs. infected)

In patients without wound infection, the mean PCT levels increased from 0.137 (SD = 0.141) preoperatively to 0.195 (SD = 0.187) on postoperative day 1 and further to 0.257 (SD = 0.303) on postoperative day 5. The p-value for the change in PCT levels over time in this group was 0.047, indicating a statistically significant difference. Conversely, in patients with wound infection, the mean PCT levels were higher at all time points, starting from 1.028 (SD = 0.568) preoperatively, slightly increasing to 1.088 (SD = 0.6266) on postoperative day 1 and reaching 1.160 (SD = 0.5746) by postoperative day 5. However, the p-value of 0.202 suggests that the changes in PCT levels over time in this group were not statistically significant (Table 4 and Figure 4).

**Table 4:** Multiple comparisons of the mean procalcitonin levels at different time intervals based on wound infection using repeated measures ANOVA *Significant (p < 0.05), SD: standard deviation

Wound infection	Time interval	N	Minimum	Maximum	Mean	SD	p-value
Absent	Preoperative	31	0.012	0.540	0.137	0.141	0.047*
	Postoperative day 1	31	0.014	0.631	0.195	0.187	
	Postoperative day 5	31	0.031	1.510	0.257	0.303	
Present	Preoperative	9	0.397	2.170	1.028	0.568	0.202
	Postoperative day 1	9	0.396	2.200	1.088	0.626	
	Postoperative day 5	9	0.410	1.990	1.160	0.574	

In patients without wound infection, the mean difference in PCT levels between the preoperative and postoperative day 1 interval was -0.058, with a p-value of 0.02, which is statistically significant. The comparison between preoperative and postoperative day 5 showed a mean difference of -0.120 with a p-value of 0.025, also indicating a significant trend. Lastly, the difference between postoperative day 1 and day 5 had a mean difference of -0.062 with a p-value of 0.229, indicating no statistically significant change. For patients with wound infection, the mean differences between preoperative and postoperative day 1, preoperative and postoperative day 5, and postoperative day 1 and day 5 were -0.059 (p = 0.463), -0.131 (p = 0.523), and -0.072 (p = 0.949) respectively, all of which were not statistically significant (Table 5).

**Table 5:** Comparison of the procalcitonin levels between different time intervals based on wound infection using post hoc least significant difference (LSD) *Significant (p < 0.05)

	Wound Infection			
	Absent (31)		Present (9)	
	Mean difference	p-value	Mean difference	p-value
Preoperative vs. postoperative day 1	-0.058	0.020*	-0.059	0.463
Preoperative vs. postoperative day 5	-0.120	0.025*	-0.131	0.523
Postoperative day 1 vs. day 5	-0.062	0.229	-0.072	0.949

## Discussion

Fracture-related infections can lead to complications such as delayed union or non-union, often requiring amputation or resulting in functional loss. These infections significantly impact the quality of life and contribute to increased healthcare costs due to the necessity for additional surgical procedures, extended hospital stays, and higher readmission rates. Prevention and early, accurate identification of infections is crucial to avoid such adverse outcomes. PCT, a precursor of the calcitonin hormone, is produced by the thyroid gland C cells. Elevated PCT levels are often associated with bacterial infections, suggesting its potential utility as a marker for early detection of infections, including sepsis. However, PCT levels can also rise due to burns, severe trauma, or surgical interventions, even in the absence of infection [17].

In this study, the mean age of participants was 42.26 ± 16.62 years, with the majority 10 (24.39%) falling within the 36- to 45-year age group. In contrast, Spoto et al. reported a mean age of 67 ± 12 years in their study population. Male predominance was evident in this study, with 35 (87.5%) of patients being male, aligning with Spoto et al., who also observed a male majority (54%) [18].

RTA was the leading cause of injury, accounting for 21 (52.5%), followed by falls from height for seven (17.5%), machine cut injuries for five (12.5%), falls of heavy objects for four (10%), and other causes such as explosions, assaults, and sickle cut injuries for each one (2.5%). Similar findings were noted in studies by Sadat-Ali et al., who reported motor vehicle accidents in 40.4% of cases, and Konbaz et al., who documented RTAs in 62.4% of their cohort [19,20].

The tibia was the most commonly fractured bone, accounting for 14 (35%), followed by tibia and fibula fractures for eight (20%). These results are consistent with Alhawas et al., who found that tibial fractures accounted for 26.1% of cases [21].

GA type 3B fractures were observed in 18 (45%), with an infection rate of 67%. Hertel et al. emphasised that early reconstruction within 24 hours significantly improved outcomes in GA type 3B and 3C tibial fractures, particularly concerning infection and bone union rates [22].

The mean preoperative PCT level for all open fractures in this study was 0.33 ± 0.46, which rose to 0.39 ± 0.49 on postoperative day 1 and 0.45 ± 0.53 on day 5. This steady increase during the perioperative period suggests that surgical trauma influences PCT levels. These findings are consistent with those of Abu Elyazed et al., who reported significant increases in PCT levels postoperatively [23]. Statistically significant differences were noted between preoperative and postoperative day 1 levels (p = 0.006) and between preoperative and postoperative day 5 levels (p = 0.008). However, the difference between day 1 and day 5 PCT levels was not significant (p = 0.132), indicating that surgical intervention may elevate PCT levels irrespective of infection. In trauma patients with an Injury Severity Score (ISS) greater than 16, Billeter et al. found that PCT levels were significantly higher on days 1 to 5 after injury, with a peak on day 1 [24].

In this study, nine patients (21.95%) developed wound infections. Among these patients, the mean preoperative PCT level was 1.02 ± 0.56, increasing to 1.08 ± 0.62 on day 1 and 1.16 ± 0.57 on day 5. In contrast, for patients without infections, mean preoperative PCT levels were 0.13 ± 0.14, increasing to 0.19 ± 0.18 on day 1 and 0.25 ± 0.3 on day 5. These findings indicate that PCT levels gradually increased in all patients with open fractures, and a preoperative PCT value >0.5 ng/mL predicted postoperative wound infection. In contrast, values <0.1 ng/mL excluded the likelihood of infection.

*Escherichia coli* and *Enterococcus faecalis* were isolated among infected fractures in 22.2% of cases. The synergistic interaction between these organisms, as seen in this study, was also documented by Montravers et al. and Lavigne et al. [25,26].

In non-infected patients, PCT levels steadily increased from preoperative to postoperative day 1 and day 5 but never exceeded 0.5 ng/mL. Although higher PCT levels in infected patients were predictive of infection, the steady perioperative rise in non-infected cases was likely attributable to surgical trauma rather than infection. These findings align with Yasmin et al., who noted systemic complications in patients with postoperative PCT levels >0.5 ng/mL [27]. Similarly, Amanai et al., in a study of 114 patients, reported significant differences in PCT levels between infected and non-infected groups, supporting PCT’s utility as a marker for postoperative infection [28].

Study limitations

Results from a single-centre study may not be generalisable to broader populations. Also, the short duration of the study with a smaller number of cases may limit the scope of the study.

## Conclusions

This study aimed to evaluate the utility of PCT as a predictor of postoperative infections in open fractures. Findings revealed that elevated preoperative PCT levels were significantly associated with the development of postoperative wound infections. While both infected and non-infected groups exhibited a steady perioperative rise in PCT levels, the infected group displayed significantly higher values, which were linked to the occurrence of wound infections. This differentiation highlights the potential of PCT to distinguish between infection-driven increases and those caused by surgical trauma in non-infected cases. Thus, PCT may serve as a valuable biomarker for the early detection of postoperative infections in open fractures. Additionally, PCT could complement bacteriological data to assess the severity and prognosis and guide therapeutic decisions for sepsis. Further research with larger sample sizes is recommended to validate these findings.
